# Immunomodulatory effects of nicotine on interleukin 1β activated human astrocytes and the role of cyclooxygenase 2 in the underlying mechanism

**DOI:** 10.1186/s12974-016-0725-1

**Published:** 2016-09-29

**Authors:** Priya Revathikumar, Filip Bergqvist, Srividya Gopalakrishnan, Marina Korotkova, Per-Johan Jakobsson, Jon Lampa, Erwan Le Maître

**Affiliations:** Department of Medicine, Unit of Rheumatology, Center for Molecular Medicine (CMM), Karolinska Institute, Karolinska University Hospital, Stockholm, Sweden

**Keywords:** Nicotine, Astrocytes, Prostaglandins, Cyclooxygenase 2, Cholinergic immune regulation, Alpha7 nicotinic acetylcholine receptor, Neuroinflammation

## Abstract

**Background:**

The cholinergic anti-inflammatory pathway (CAP) primarily functions through acetylcholine (ACh)-alpha7 nicotinic acetylcholine receptor (α7nAChR) interaction on macrophages to control peripheral inflammation. Interestingly, ACh can also bind α7nAChRs on microglia resulting in neuroprotective effects. However, ACh effects on astrocytes remain elusive. Here, we investigated the effects of nicotine, an ACh receptor agonist, on the cytokine and cholinesterase production of immunocompetent human astrocytes stimulated with interleukin 1β (IL-1β) in vitro. In addition, the potential involvement of prostaglandins as mediators of nicotine was studied using cyclooxygenase 2 (COX-2) inhibition.

**Methods:**

Cultured human fetal astrocytes were stimulated with human recombinant IL-1β and treated simultaneously with nicotine at different concentrations (1, 10, and 100 μM). Cell supernatants were collected for cytokine and cholinesterase profiling using ELISA and MesoScale multiplex assay. α7nAChR expression on activated human astrocytes was studied using immunofluorescence. For the COX-2 inhibition studies, enzyme activity was inhibited using NS-398. One-way ANOVA was used to perform statistical analyses.

**Results:**

Nicotine treatment dose dependently limits the production of critical proinflammatory cytokines such as IL-6 (60.5 ± 3.3, %inhibition), IL-1β (42.4 ± 1.7, %inhibition), and TNF-α (68.9 ± 7.7, %inhibition) by activated human astrocytes. Interestingly, it also inhibits IL-8 chemokine (31.4 ± 8.5, %inhibition), IL-13 (34.243 ± 4.9, %inhibition), and butyrylcholinesterase (20.8 ± 2.8, %inhibition) production at 100 μM. Expression of α7nAChR was detected on the activated human astrocytes. Importantly, nicotine’s inhibitory effect on IL-6 production was reversed with the specific COX-2 inhibitor NS-398.

**Conclusions:**

Activation of the cholinergic system through α7nAChR agonists has been known to suppress inflammation both in the CNS and periphery. In the CNS, earlier experimental data shows that cholinergic activation through nicotine inhibits microglial activation and proinflammatory cytokine release. Here, we report similar anti-inflammatory effects of cholinergic activation on human astrocytes, at least partly mediated through the COX-2 pathway. These results confirm the potential for cholinergic neuroprotection, which is looked upon as a promising therapy for neuroinflammation as well as neurodegenerative diseases and stroke. Our data implicates an important role for the prostaglandin system in cholinergic regulatory effects.

## Background

Inflammation plays a crucial role in our self-defense and is orchestrated by a series of well-characterized immune responses to eliminate the invading pathogen. While the initiation of an immune response is of paramount importance to keep life-threatening diseases at bay, a failure in resolving inflammation and restoring homeostasis after the threat is eliminated ultimately results in chronic inflammation and puts the organism in jeopardy. Thus, inflammation is a double-edged sword and needs to be closely monitored by the innate anti-inflammatory mechanisms. One such mechanism, which was discovered few decades ago and is now looked upon as a promising therapy to treat chronic inflammatory diseases, is called the “cholinergic anti-inflammatory pathway” (CAP) (for a review, see [1]).

The CAP is believed to be a highly conserved mechanism in which the central nervous system (CNS) exhibits its anti-inflammatory role in the periphery through the efferent vagus nerve in a discrete and localized manner where inflammation had typically originated. Acetylcholine (ACh), the principal neurotransmitter of CAP released by splenic T lymphocytes in response to the efferent vagus nerve activation, acts on specific α7 nicotinic acetylcholine (α7nACh) receptors on activated macrophages to inhibit the release of proinflammatory cytokines, thereby limiting peripheral inflammation [1]. On the other hand, afferent activation of the vagus nerve has been shown to increase ACh release in the brain, mediated by adrenergic activation of the central cholinergic network [2–4]. One of the important effects for the centrally released ACh is to specifically bind to α7nACh receptors on microglia, thereby promoting anti-inflammatory and neuroprotective effects in CNS [5]. The latter has been well documented [6–8], and different agonists for nicotinic acetylcholine receptors (nAChRs; α7 subtype in particular) are now in clinical trials for treatment of neurodegenerative diseases.

nAChRs are widely expressed by cells in the central and peripheral nervous systems, immune system, and other peripheral tissues. In the CNS, both neuronal and non-neuronal cells that include astrocytes, microglia, oligodendrocytes, and endothelial cells express α7nAChR [9]. While nAChRs, in general, are known to participate in memory, learning, locomotion, and many other physiological functions, activation of α7nAChRs have been linked to neuroprotection and neuron survival [10, 11]. Interestingly, mechanistic studies on the downstream effects of nicotine-α7nAChR interaction in rat microglia revealed an up-regulation of cyclooxygenase 2 (COX-2) and prostaglandin E_2_ (PGE_2_) synthesis, implicating prostaglandins (PGs) as one of the important mediators of cholinergic effects in the CNS [6]. However, the magnitude of these effects, and their impact on other resident CNS cells such as astrocytes, is largely unknown. PGE_2_ is a bioactive lipid molecule produced by the action of cyclooxygenases (COX-1/2) and terminal synthases (microsomal prostaglandin E synthases (mPGES-1 and mPGES-2) and cytosolic prostaglandin E_2_ synthase (cPGES)) on arachidonic acid released from the cell membrane by phospholipase A_2_ following physiological or inflammatory stimuli.

Astrocytes are the most abundant glial cells in the CNS and also confer important roles in neuroinflammation. These resident cells produce a plethora of cytokines such as interleukin 1β (IL-1β) and interleukin 6 (IL-6) which are implicated in several neurodegenerative diseases [12, 13]. In addition, astrocytes also happen to be a major source of one of the key ACh-hydrolyzing enzymes, butyrylcholinesterase (BuChE). Increased BuChE expression is strongly correlated with activation of astrocytes [14]. However, the role of the other ACh-hydrolyzing enzyme acetylcholinesterase (AChE) with respect to astrocytes remains unclear. In fact, both CSF and plasma BuChE and AChE levels are elevated in many clinical ailments [15]. On the contrary, astrocytes can also secrete anti-inflammatory cytokines such as transforming growth factor β (TGFβ), which in turn can limit microglial activation during inflammation [16, 17]. Intriguingly, astrocytes have been shown to express α7nACh receptors whose activation mediates neuroprotective effects (against neuroinflammation) following nicotine administration [18]. It is important to note that these observations were made in mice and hence one can question their relevance to humans. Thus, the need to investigate and confirm nicotine’s effects on activated human astrocytes demands attention.

In the present study, we aimed to activate human fetal astrocytes with recombinant IL-1β and study the effects of nicotine on the proinflammatory cytokine and cholinesterase (AChE and BuChE) production. In addition, downstream mechanisms of nicotine’s action were studied with a focus on the prostaglandin pathway, earlier known from peripheral immune cells to be involved in cholinergic immunomodulatory effects.

## Methods

### Human fetal astrocytes culture and treatment

Cryopreserved primary human fetal astrocytes (approx. 1 million cells, SC1800, ScienCell Research Laboratories ﻿purchased from 3H Biomedical AB, Sweden) were cultured in poly-l-lysine-coated T-175 flasks as per supplier’s instructions. Cells were incubated in a humidified incubator with 5 % CO_2_ at 37 °C, and astrocyte medium supplemented with fetal bovine serum, astrocyte growth supplement, and penicillin-streptomycin (SC1801, ScienCell Research Laboratories purchased from 3H Biomedical AB, Sweden) was changed once every 2 days till the cells reached 80–90 % confluency. Later, the cells were split using trypsin-EDTA and seeded in poly-l-lysine-coated 6 well plates (20,000 cells/well) or chamber slides (8000 cells/well). One day prior to treatment, the complete medium was replaced with serum-starved medium. The next day, cells were stimulated with IL-1β (10 ng/ml, PHC0815, Invitrogen) and nicotine (1, 10, and 100 μM, N3879, Sigma) simultaneously. In the COX-2 inhibition studies, NS-398 (1 or 10 μM, Cayman Chemical) was added to the cells 30 min prior to the treatment mentioned above. After 20 h, cell supernatants were collected, centrifuged, and stored at −20 °C for further use. The cells on the chamber slides were formaldehyde- or acetone fixed for glial fibrillary acidic protein (GFAP) and α7nAChR staining, respectively, air dried, and stored at −80 °C. Cell cultures were tested negative for mycoplasma contamination.

### Immunofluorescence for characterizing astrocytes and co-localization with α7nACh receptor

Formaldehyde-fixed slides were taken out from −80 °C storage and were washed with PBS-saponin. Following that, the slides were incubated overnight at 4 °C with primary mouse monoclonal antibodies against GFAP (8152, Cell Signaling) tagged with Alexa Fluor 594 in a 1:50 dilution containing 3 % normal human serum. For assessing co-localization of GFAP and α7nACh receptor expression, in addition to GFAP primary antibodies, the cells were also incubated overnight with rabbit polyclonal against α7nACh receptor (1:500, ab10096, Abcam). Later, the cells were treated with biotinylated goat anti-rabbit (1:1600, Vector Laboratories) for 30 min. Then, they were incubated with streptavidin antibodies conjugated with Alexa Fluor 488 (1:1000) for 45 min. The slides were later rinsed, incubated with 4′,6-diamidino-2-phenylindole (DAPI, 1:2000) for 1 min at room temperature and mounted using PBS-glycerol. The slides were examined under a microscope (Leica Microsystems, Cambridge, UK), and images were taken at ×20 or ×40 magnification.

### Cytokine measurement using sandwich enzyme-linked immunosorbent assay

The cytokine concentrations in the culture supernatants were measured using human IL-6 (DuoSet, DY206) and IL-8 (DuoSet, DY208) from R&D Systems. The detection range for IL-6 and IL-8 cytokine measurement is 9.38–600 and 31.20–2000 pg/ml, respectively. Microtiter plates were incubated with 100 μl of capture antibody (mouse anti-human IL-6 or IL-8) per well overnight at room temperature. Next, the plates were washed with PBS-Tween as per instructions and incubated for an hour with blocking solution. Following that, 100 μl of the samples and standards were added to the wells and incubated at room temperature. After 2 h, the wells were washed and incubated with detection antibodies (biotinylated goat anti-human IL-6 or IL-8) for 2 h. Later, streptavidin-HRP and substrate solutions were added and incubated as per protocol. Finally, the plates were read at 450 nm with the correction wavelength at 570 nm. The samples were diluted accordingly to comply with the detection limits of the assay kits.

### Measurement of Th1 and Th2 cytokines

We measured interferon (IFN)-γ, IL-1β, IL-2, IL-4, IL-6, IL-8, IL-10, IL-12p70, IL-13, and TNF-α levels in the cell supernatants using the ultrasensitive MesoScale Discovery (MSD) MULTI-SPOT assay (Human Proinflammatory Panel 1, K15049D, MSD). The detection range is 0.36–1460, 0.12–495, 0.36–1460, 0.05–198, 0.19–767, 0.12–495, 0.08–334, 0.10–421, 0.11–466, and 0.08–312 pg/ml, respectively. This is a sandwich immunoassay where 50 μl of the samples and standards are first added, as technical duplicates, to the pre-coated plates and incubated overnight at 4 °C. Next, the plates were washed to remove unbound substances and incubated with electrochemiluminescent conjugated detection antibodies for 2 h at room temperature. The plates are then treated with MSD buffer and read using a MSD instrument (SECTOR Imager 2400). The standards for different cytokines vary in their range and are hence tailored according to the possible quantities that might be detected for an individual cytokine.

### Immunohistochemistry for α7nACh receptor expression

Acetone-fixed human astrocytes, treated with IL-1β and nicotine, were stained for the expression of the α7nACh receptor. The slides were first washed in PBS-0.1 % saponin solution at room temperature. Then, endogenous hydrogen peroxidase enzyme activity and avidin and biotin binding sites were blocked using 1 % hydrogen peroxide and avidin-biotin blocking kit, respectively. The slides were then blocked with 3 % normal goat serum to avoid any non-specific binding. The slides were washed three times lasting 3 min each. The primary antibody against the α7nACh receptor (1:1200, monoclonal rat anti-human, ab24644, abcam) containing 3 % normal human serum was added to the slides and incubated overnight at 4 °C. The next day, the slides were washed and incubated with 1 % normal goat serum followed by incubation with secondary antibodies (biotinylated anti-rat IgG raised in goat, Vector Laboratories) for 30 min at room temperature. The signal for the protein expression was developed using the avidin-biotin complex (ABC complex, Vector Laboratories) and DAB substrate kit (3,3′-diaminobenzidine and H_2_O_2_, Vector Laboratories). Finally, the slides were mounted using PBS-glycerol and viewed under a fluorescence microscope (Leica Microsystems, Cambridge, UK). Photomicrographs (×20 or ×40) of the labeled regions were obtained using the Leica Application Suite (LAS version 4.4). Rat IgG antibodies were used as isotype control.

### Enzyme immunoassay for PGE_2_ quantification

Prostaglandin E_2_ levels in the cell supernatants were measured using a forward sequential competitive binding technique in which the analyte competes with horseradish peroxidase (HRP)-conjugated PGE_2_ to bind to a specific number of sites on a mouse monoclonal antibody (Prostaglandin E2 Parameter Assay Kit, KGE004B, R&D Systems). First, the samples, standards, and controls were allowed to bind to the antibody pre-coated on the plate for an hour. After the incubation, the HRP-conjugated PGE_2_ was added to the wells where it binds to the remaining binding sites on the plate. A substrate solution, to quantify the bound HRP activity, was added to all wells following a wash that removed unbound substances. The absorbance of the color, thus developed, was measured at 450 nm.

### Prostaglandin analysis by Liquid chromatography tandem mass spectrometry (LC-MS/MS)

Prostaglandins in cell supernatants were extracted and analyzed according to Idborg et al. [19]. Working in duplicates, 450 μl of samples were spiked with 50 μl deuterated internal standards of 6-keto-PGF_1α_, PGF_2α_, PGE_2_, PGD_2_, TxB_2_, and 15-deoxy-Δ12,14PGJ_2_ (Cayman Chemical Company) and made acidic with 500 μl 0.2 % formic acid (FA) in Milli-Q. The samples were loaded on Oasis HLB 1 cc 30 mg cartridge (Waters Corporation, MA, USA) that had been preconditioned with methanol and 0.05 % FA in Milli-Q. Prostaglandins were eluted in 1 ml methanol and evaporated to dryness under vacuum. Samples were stored at −20 °C until resuspended in 50 μl 7 % acetonitrile prior to analysis with LC-MS/MS. The extracted prostaglandins were quantified using a triple quadrupole mass spectrometer (Acquity TQ Detector, Waters Corporation) equipped with a Waters 2795 HPLC (Waters Corporation). Separation was performed on a 100 × 2.0 mm Synergi 2.5 μm Hydro-RP 100 Å column (Phenomenex, CA, USA) with a 45-min stepwise linear gradient (10–90 %) of 0.05 % FA in acetonitrile as mobile phase B and Milli-Q as mobile phase A. Individual prostaglandins were detected in multiple reaction monitoring mode. Data were analyzed using MassLynx Software, version 4.1, with internal standard calibration and quantification to external standard curves.

### Measurement of AChE and BuChE in cell supernatants

Protein concentration of AChE and BuChE in the astrocyte culture supernatants was measured using quantitative sandwich ELISA technique employed in human acetylcholinesterase (DACHE0) and human butyrylcholinesterase (DBHE0) quantikine ELISA kits (R&D Systems). The detection ranges are 125–8000 pg/ml for AChE and 0.156–10 ng/ml for BuChE. Briefly, undiluted samples and standards were added to the microplate strips pre-coated with monoclonal antibodies specific for human AChE and BuChE and incubated for 2 h at room temperature. Following this, plates were washed as per manufacturer’s protocol and incubated with conjugate for two more hours. After the last wash, the plates were incubated with the substrate solution and the plates were read after 30 min at 450 nM, and the reference wavelength was set to 540 nM.

### Statistics

All experiments were repeated at least three to five times unless otherwise indicated. Samples were run in duplicates for all our assays, and corresponding data are represented as mean ± SEM. Statistical analyses were performed using one-way ANOVA with unpaired Student’s *t* test as mentioned, and the significance was set at *p* value <0.05. All the statistical tests were done using GraphPad Prism 6.0 software.

## Results

### Expression of glial fibrillary acidic protein in human astrocyte cultures

**Fig. 1:**
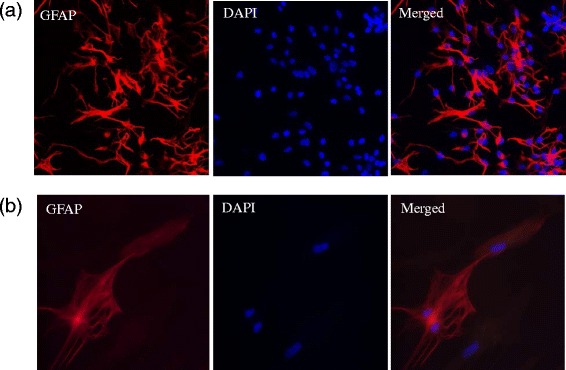
Characterization of cultured human cortical astrocytes using immunofluorescence. Human astrocytes were identified using GFAP monoclonal antibody (Alexa Fluor 594, *red*) and DAPI (*blue*). Fluorescence microscopy images of human astrocytes taken at **a** ×20 representing GFAP-positive (93 %) human astrocytes and **b** ×40 magnification

First, we confirmed the identity of the human fetal astrocytes by examining the expression of the most predominant astroglial marker glial fibrillary acidic protein (GFAP), an intermediate filament protein by using immunofluorescence. Our astrocyte cultures displayed strong GFAP expression both in the resting and activated states as shown in Fig. 1. We quantified the GFAP-positive cells to be approximately 93 % of the total cells. Our results thus confirmed the manufacturer’s claim that more than 90 % of the cells were positive for GFAP expression. The possibility of non-specific staining was ruled out using an appropriate negative control.

### Kinetics of IL-6 release by human astrocytes activated with IL-1β

**Fig. 2:**
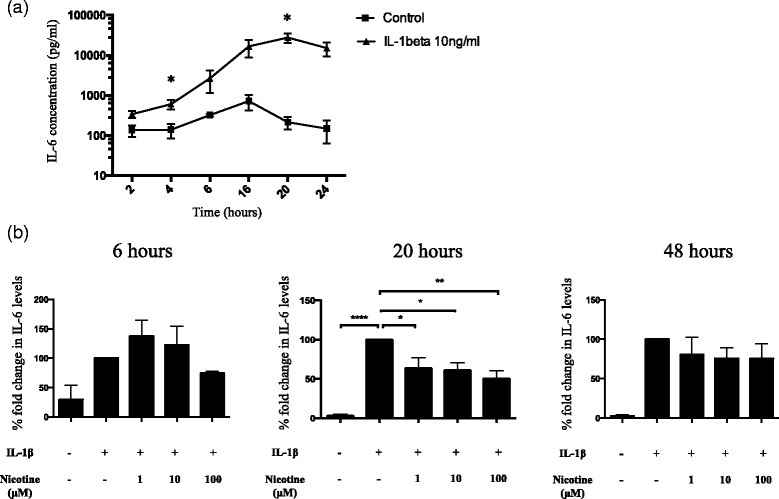
Kinetics and inhibition of IL-6 release by human astrocytes in response to IL-1β and nicotine treatment. **a** IL-6 concentration in the cell culture supernatants collected at 2, 4, 6, 16, 20, and 24 h was assessed using sandwich ELISA. Values are represented as mean ± SEM of three independent experiments performed in duplicates. Statistical analyses was done using Student’s *t* test.(*p < 0.05). **b** Cell culture supernatants of activated human astrocytes treated with nicotine at different concentrations for 6, 20, and 48 h were analyzed for IL-6 levels using sandwich ELISA. Samples were run as duplicates, and values are represented as mean ± SEM from at least three independent experiments (*n* = 3 for 6 h, *n* = 6 for 20 h, and *n* = 4 for 48 h). Data is presented in terms of fold change compared to the IL-6 level following IL-1β treatment. Following 20-h nicotine treatment, IL-6 protein levels were downregulated dose dependently in the cell supernatants. Statistical analyses was performed using one-way ANOVA (****p < 0.0001, **p < 0.01, *p < 0.05)

Next, human astrocytes were stimulated with IL-1β (10 ng/ml) for 0, 2, 4, 6, 16, 20, and 24 h. Measurement of IL-6 release in the cell supernatants revealed that IL-1β increased IL-6 levels fourfold compared to the control as early as 4 h after treatment (604.8 ± 159.2 vs 139.6±55.9 pg/ml, *p* = 0.05) and steadily increased manyfold thereafter. The peak for IL-6 production was reached at 20 h of IL-1β treatment (27,698.5 ± 7215.96 vs 214.96±71.94 pg/ml, *p* < 0.05) (Fig. 2a).

### Nicotine treatment significantly inhibits IL-6 release by human astrocytes

We incubated the IL-1β activated human astrocytes with nicotine at various concentrations (1, 10, and 100 μM) for 6, 20, and 48 h respectively. As mentioned earlier, IL-1β was found to induce significant levels of IL-6 release in the supernatant compared to the control. Interestingly, nicotine dose dependently downregulated IL-6 production at 20 h. The inhibition was up to 49.5 ± 9.5 % at 100 μM nicotine concentration as displayed in Fig. 2b. In addition, nicotine alone did not exert any effect on the IL-6 release by human astrocytes (data not shown).

### Effects of nicotine on Th1/Th2 cytokine production by human astrocytes

**Fig. 3:**
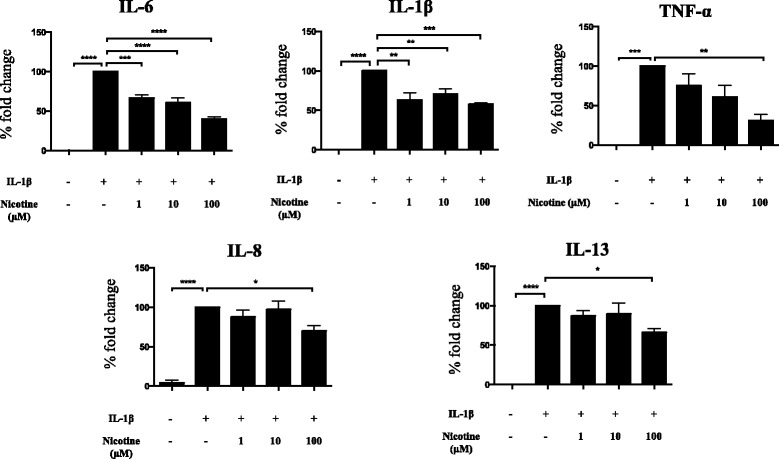
Modulating effects of nicotine on multiple cytokines during neuroinflammation. Activated human astrocytes were treated with nicotine at 1, 10, and 100 μM for 20 h, and their supernatants were collected. Cytokine profiling related to Th1/Th2 immune response was performed using multiplex cytokine ELISA and human IL-8 sandwich ELISA. Samples were run as duplicates, and values are represented as mean ± SEM from at least three independent experiments (*n* = 3 for multiplex ELISA and *n* = 6 for sandwich ELISA). Data is presented in terms of fold change compared to cytokine levels following IL-1β treatment. Nicotine treatment significantly reduced the production of IL-6, TNF-α, IL-1β, IL-13, and chemokine IL-8. Statistical analyses was performed using one-way ANOVA (****p < 0.0001, **p < 0.01, *p < 0.05)

Following the limiting effects of nicotine on IL-6 release by human astrocytes after incubation for 20 h, we aimed to measure the levels of various other cytokines associated with IL-1β mediated inflammation. Using a multiplex assay, we reconfirmed the effects on IL-6 release. Interestingly, as illustrated in Fig. 3, we discovered that nicotine had similar immunomodulatory effects on TNF-α (68.9 ± 7.7, %inhibition at 100 μM nicotine treatment, *p* < 0.05), IL-1β (42.4 ± 1.7, %inhibition at 1, 10, and 100 μM nicotine treatment, *p* < 0.01), IL-8 (31.4 ± 8.5, %inhibition at 100 μM nicotine treatment, *p* < 0.05), and IL-13 (34.243 ± 4.9, %inhibition at 100 μM nicotine treatment, *p* < 0.05) cytokines. On the contrary, nicotine did not affect the production of the other cytokines measured including IFN-γ, IL-2, IL-4, IL-10, and IL-12p70 which were, in fact, secreted at low levels after IL-1β stimulation (data not shown).

### Nicotine decreases BuChE protein release from activated human astrocytes

**Fig. 4:**
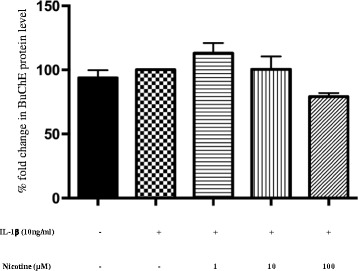
Butyrylcholinesterase levels in cell culture supernatants of IL-1β activated human astrocytes following nicotine treatment. Both untreated and IL-1β-activated human astrocytes released similar BuChE protein levels into their supernatants following 20-h incubation. Nicotine dose dependently inhibited BuChE protein release by activated human astrocytes. Samples were run as duplicates, and values are represented as mean ± SEM of four independent experiments. Data is presented in terms of fold change compared to BuChE levels following IL-1β treatment

We measured acetylcholinesterase (AChE) and butyrylcholinesterase (BuChE) protein levels in IL-1β activated astrocyte supernatants following nicotine treatment (1, 10, and 100 μM) for 20 h. BuChE was detectable in all treatment supernatants as shown in Fig. 4. Human astrocytes released BuChE into the supernatant even under normal conditions. Nicotine treatment reduced BuChE levels at 100 μM (79.3 ± 2.8, % fold change compared to IL-1β treatment alone). Moreover, we observed a tendency of dose-dependent nicotine-induced reduction of BuChE levels. IL-1β alone did not have any significant effects on BuChE protein levels when compared to the control. AChE was not detected in any of the cell culture supernatants.

### Expression of α7nAChR in activated human astrocytes

**Fig. 5:**
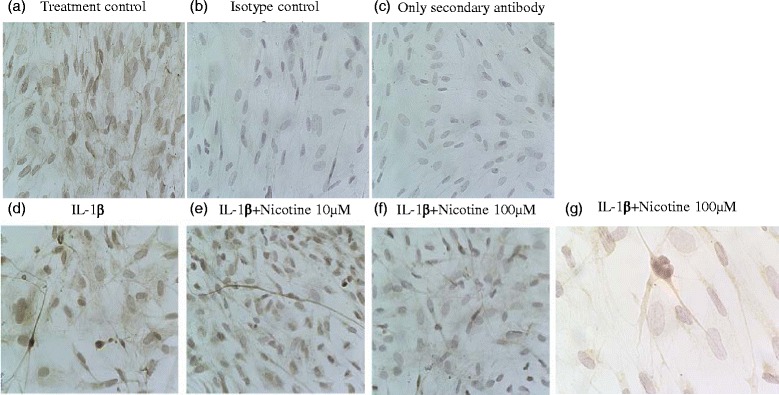
Expression of α7 nicotinic acetylcholine receptor on activated human astrocytes. Human astrocytes grown on chamber slides were incubated with IL-1β and nicotine for 20 h and later fixed in paraformaldehyde for immunohistochemistry. Positively stained cells appear *brown* in the images. Cells both **a** untreated and **d**–**f** treated with nicotine displayed specific staining for α7nACh receptor protein expression whereas cells stained with **b** unspecific antibody raised in rat (isotype) and **c** goat anti-rat (secondary antibody) alone displayed no specific staining. Representative images were taken at ×25 (**a**–**f**) and ×40 (**g**) magnification under the microscope

**Fig. 6:**
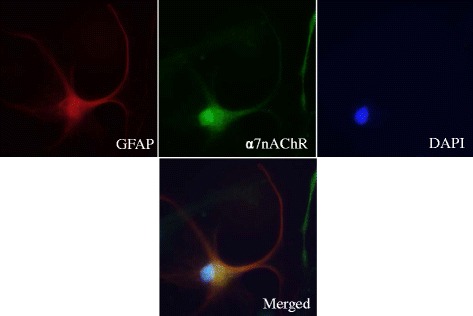
Co-localization of α7 nicotinic acetylcholine receptor on activated human astrocytes treated with nicotine. Double immunofluorescence images showing the expression of astroglial marker GFAP (*red*), α7nACh receptor (*green*), and nucleus (*blue*). Representative images were taken at ×40 magnification under the microscope

Next, we studied the expression of the α7nACh receptor in IL-1β-activated human astrocytes treated with nicotine. We could confirm astrocyte expression of the α7nACh receptor protein with or without nicotine at various concentrations as shown in Fig. 5. Isotype and secondary antibodies alone did not show any positive signal reducing the possibility of non-specific binding. Furthermore, using immunofluorescence, we could demonstrate that nicotine-activated GFAP-positive human astrocytes displayed a strong expression of α7nACh receptor (Fig. 6).

### Nicotine treatment promotes human astrocytes to release more prostaglandin E_2_

**Fig. 7:**
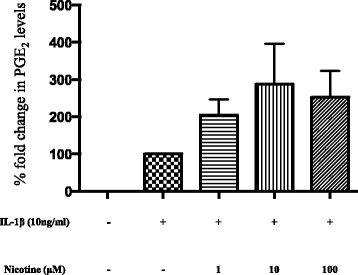
Prostaglandin E_2_ synthesis in human astrocytes induced by nicotine treatment. Cell culture supernatants were assayed for PGE_2_ levels using enzyme immunoassay (EIA). IL-1β-induced PGE_2_ synthesis in human astrocytes and treatment with nicotine increased it further dose dependently. Samples were run as duplicates, and PGE_2_ levels following various treatments are represented as mean ± SEM from four independent experiments. Data is presented in terms of fold change compared to cytokine levels following IL-1β treatment

Known for its close association with IL-1β-initiated inflammation and human astrocytes, PGE_2_ production was quantified in the culture supernatants accordingly. Intriguingly, IL-1β-induced PGE_2_ release by activated human astrocytes further tends to increase after incubation with nicotine (204.88 ± 41.77 at 1 μM, 287.15 ± 108.36 at 10 μM, and 251.94 ± 71.09 at 100 μM; % fold increase compared to IL-1β treatment alone, Fig. 7). Nicotine alone did not induce PGE_2_ above detectable levels (data not shown).

### COX-2 inhibition reverses nicotine effects on human astrocytes

**Fig. 8:**
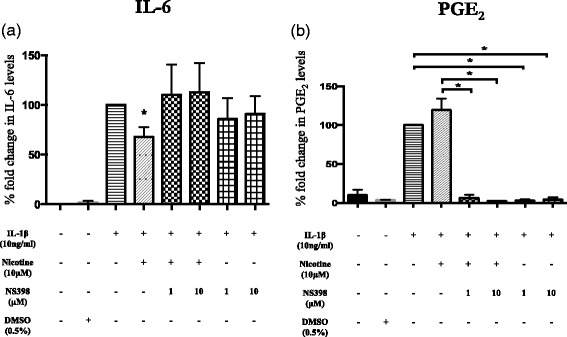
Role of COX-2 during the effects of nicotine on IL-6 release from activated human astrocytes. **a** Inhibition of cyclooxygenase 2 activity by using NS-398 (1 and 10 μM) reversed the effects of nicotine on IL-6 release from activated human astrocytes. The COX-2 inhibitor failed to affect IL-1β-induced IL-6 production on its own. **b** Cell culture supernatants were analyzed for prostaglandin profiling using LC-MS/MS. IL-1β induced high levels of PGE_2_ production that further increased with nicotine treatment. PGE_2_ synthesis was completely blocked in the presence of NS-398 at both concentrations. Samples were run as duplicates, and values are represented as mean ± SEM of (a) five and (b) three independent experiments. Data is presented in terms of fold change compared to cytokine levels following IL-1β treatment. Statistical analyses was performed using Students *t* test and one-way ANOVA (*p < 0.05)

Then, we investigated the effects of COX-2 inhibition on the immunomodulatory effects of nicotine on IL-6 cytokine production in activated human astrocytes. COX-2 activity was blocked using NS-398 at 1 and 10 μM. Cells exposed to NS-398 failed to reduce the IL-6 release in the presence of nicotine (10 μM) as shown in Fig. 8a. Furthermore, incubation with only NS-398 did not exhibit any effects on the astrocytes. LC-MS/MS studies on the cell culture supernatants were performed to measure the PGE_2_ production following NS-398 treatment, and the results are demonstrated in Fig. 8b. In accordance with our previous observations, nicotine tends to increase IL-1β-induced PGE_2_ release (119.66 ± 14.4, % fold change compared to IL-1β treatment alone). In addition, a complete inhibition of PGE_2_ production was observed in the cells treated with NS-398. On the contrary, other members of the prostanoid family were at undetectable levels. Dimethylsulfoxide (DMSO) used to dissolve NS-398 did not have any effects on its own. Nicotine treatment alone did not induce PGE_2_ (data not shown).

## Discussion

**Fig. 9:**
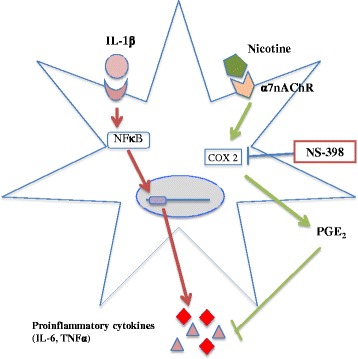
Schematic representation of the immunomodulatory effects of nicotine on human fetal astrocytes. During neuroinflammation, proinflammatory cytokines such as IL-1β stimulate the production of NF-κB-dependent cytokines such as IL-6 and TNF-α. In our study, we observed that nicotine treatment inhibits the production of such inflammatory cytokines possibly through the COX-2 pathway to restore homeostasis. Nicotine binds to its α7nACh receptor on astrocytes and increases a COX-2-dependent PGE_2_ level that seems to be a crucial mechanism

Cholinergic signaling comprises a network of effects both on resident cells in the CNS and immune cells entering the brain. Activation of the central cholinergic system, for example, by afferent electrical vagus nerve stimulation [2, 3], was earlier shown to exhibit neuroprotective effects mediated by α7nAChR on activated microglia [5, 11]. Potential immunoregulatory cholinergic systems on other immunocompetent cells in the CNS such as astrocytes remain elusive. In the present study, we focused on the potential effects of CAP activation through the administration of cholinergic agonists on human astrocytes, which are immunocompetent effector cells capable of antigen presentation and cytokine and chemokine production during CNS inflammation [12]. We found that incubation with nicotine has immunosuppressive effects on the production of proinflammatory cytokines in IL-1β-activated human fetal astrocytes. Moreover, a higher dose of nicotine limits the production of the chemokine IL-8 and tends to inhibit non-specific cholinesterase enzyme BuChE. In addition to being responsive to nicotine treatment, activated human astrocytes also expressed α7nAChR. Interestingly, the immunomodulatory effects of nicotine on astrocytes were reversed with COX-2 inhibition, suggesting prostaglandins as important mediators in this context (Fig. 9).

When testing different ways for astrocyte activation in vitro, we first found, in agreement with earlier data [20, 21], that human fetal astrocytes in contrast to mouse astrocytes failed to respond to lipopolysaccharides (LPS). However, another mediator known to exert functional effects on astrocytes is IL-1β. Elevated levels of IL-1β have been shown to induce IL-6 and TNF-α production in astrocytes thereby propagating further inflammation and resulting in neuronal death [20, 22]. As expected, activation of primary human fetal astrocytes with IL-1β induced astrogliosis defined by proliferation, morphological changes, and enhanced GFAP expression (data not shown). Furthermore, supporting the earlier studies [20, 21, 23], we could detect an IL-1β-induced increase in the levels of NF-κB-dependent inflammatory proteins IL-6 and TNF-α production.

IL-6 is a major cytokine in the CNS with both beneficial and destructive outcomes. It is well established to be actively involved in astrogliosis following an inflammatory insult [24, 25]. Furthermore, dysregulation in IL-6 expression leading to its excess in the CNS has been related to neuropathology such as neurodenegration, disruption of blood-brain barrier, angiogenesis, and elevated complement proteins [26, 27]. Importantly, IL-6-deficient mice have been shown to be resistant to experimental autoimmune encephalomyelitis (EAE) and display impaired macrophage activation in models of brain injury [28, 29]. Hence, it is likely that the increased IL-6 release by activated human astrocytes, especially in combination with TNF-α, may contribute to deleterious effects on neuronal tissue in this context.

The immunoregulatory effects of nicotine on the peripheral innate immune cells have been well established [30–32], and similar effects on glial cells during neuroinflammation were observed, both in vitro [5, 7, 33] and in vivo [34]. However, most of these observations were made in murine astrocytes and might not necessarily replicate the exact inflammatory environment in human CNS pathology. Given the fact that murine and human astrocytes respond differently to LPS [21], such differences may complicate understanding astrocyte neuroinflammatory properties and translational research in human neurodegenerative diseases.

In the present study, the peak for IL-1β-induced IL-6 production occurred after 20 h incubation and this time point was then used in the following pharmacological experiments. We could detect a strong inhibitory effect of nicotine on IL-1β-induced IL-6 release whereas nicotine alone had no such effects. This is in line with the cholinergic system not being constitutively acting as an immune regulator on non-activated cells but rather having a role for regulating excess of inflammation, as potentially induced through nearby tissue damage or other causes of local immune activation.

It is well documented that the presence of IL-1β in the environment induces astrocytes to produce IL-8, a chemokine involved in neutrophil infiltration into the CNS and pain [35]. Earlier, conflicting results have been reported on nicotine as an inducer [36, 37] or suppressor of IL-8 synthesis in other different cell types [38]. While nicotine has been shown to inhibit IL-8 synthesis in rat astrocytes [33], there are no previous data on human astrocytes. We found a strong inhibitory effect of high doses of nicotine (100 μM) on IL-8 released by activated human astrocytes. This is in line with earlier findings that nicotine pretreatment was shown to protect mice subjected to kidney ischemia injury by preventing neutrophil recruitment through attenuation of KC/CXCL1, a neutrophil chemoattractant [39]. Also, nicotine has been shown to exert similar effects on IL-8 in fibroblast-like synoviocytes derived from rheumatoid arthritis patients [40]. Altogether, the immune-suppressive effects of nicotine on IL-8 release from astrocytes in the present study hold therapeutic implications in protecting the CNS from the immunological escalation following leucocyte infiltration.

We could also observe a reduction in IL-13 levels which is mainly a Th2 response-promoting cytokine shown to have anti-inflammatory effects during neuroinflammation [41], and its downregulation in response to nicotine needs further investigation. It is also important to point out that IL-1β neither induced other Th2 cytokines such as IL-2 and IL-4 nor affected the anti-inflammatory cytokine IL-10 in our study.

AChE is primarily expressed in neurons, and in astrocytes, the expression of AChE is low, except in astrocytic tumors [42, 43]. In addition, studies have also shown AChE to exhibit very little catalytic activity in spite of AChE protein expression being detected in primary astrocytes [44, 45]. On the contrary, butyrylcholinesterase (BuChE) seems to be strongly associated with glial cells such as astrocytes [46]. In line with earlier studies, we were able to detect BuChE but not AChE protein expression in the culture supernatants when treated with IL-1β and nicotine. In addition, we observed a tendency with increasing nicotine treatment to reduce BuChE protein levels. In support of our findings, Darreh-Shori et al. [47] have published similar results showing decreased BuChE protein expression and activity with increasing acetylcholine treatment whereas no such effects were observed in the case of AChE. Though the importance of increased AChE expression during inflammation cannot be overlooked, owing to its low expression/activity in human primary astrocytes, we hypothesize that AChE might not produce any notable effects on the cytokine production of activated primary astrocytes. On the other hand, the association of elevated cortical BuChE levels with Alzheimer’s disease [46] and stroke [48] make nicotine’s inhibitory effect on BuChE activity beneficial in preventing ACh degradation, limiting astrogliosis, and eventually suppressing neuroinflammation.

Nicotine may act on several receptors, and it has to be noted that several nicotine receptor subtypes are expressed on astrocytes [33, 49]. In the present study, we believe it is likely that α7nAChR alone is mediating the immune-suppressive effects of nicotine, for a number of reasons. First, among the other subtypes of nicotine receptors, α7nAChR is the crucial nicotinic receptor mediating anti-inflammatory effects [10, 50, 51] and a key mediator of the nicotinic anti-inflammatory pathway in inflammatory diseases [32, 52]. Moreover, α7nAChR activation has been shown to protect astrocytes from oxidative stress-induced death by inhibiting the mitochondrial apoptotic pathway and thus is implicated in providing neuroprotection against several neuroinflammatory diseases [18, 53, 54]. Second, we could detect the expression of α7nAChR on the human astrocytes, and the specificity was further confirmed by double staining with GFAP, showing that all of these activated cells displayed strong expression of α7nAChR. While the beneficial effects of α7nAChR activation in combating neuroinflammation have been widely studied, the exact mechanism by which such an effect is elicited remains elusive. Mechanistic studies on the activation of α7nAChR on microglia have been shown to resolve inflammation through COX-2 and PGE_2_ up-regulation [6]. Our results from PG analysis and COX-2 inhibition studies suggest that a similar mechanism is triggered upon α7nAChR activation in astrocytes. In peripheral monocytes, Takahashi et al. [55] have shown that in vitro treatment with nicotine induced an increase in PGE_2_ levels in a COX-2-dependent manner, which in turn interacted with its EP2/4 receptors in an autocrine/paracrine fashion. The same group has also shown that activation of EP2/4 receptors led to an increase in secondary messenger cyclic adenosine monophosphate (cAMP) and protein kinase A activity, thereby inhibiting IL-18 production. Interestingly, and like nicotine, PGs have been shown to have major impact on neuroinflammation, for example, illustrated by earlier in vivo data that COX-2-deficient mice exhibited an aggravated neuroinflammatory response following intracerebroventricular LPS injection [56]. Altogether, our results thus implicate that cholinergic regulation of astrocytes, and potential neuroprotection, might be partly mediated via COX-2-dependent endogenous PGE_2_ production. In this context, it may be noted that non-steroidal anti-inflammatory drugs (NSAIDs) are small lipid molecules and are known to pass the blood-brain barrier [57]. Given the results of the present study, these drugs may exert important effects on activated resident cells of the CNS and also regulate cholinergic mechanisms. Whether these mechanisms also may affect neurosignaling or other brain functions is not yet known.

Astrocytes are well known for their constant interaction with microglia, and soluble factors released by astrocytes are known to regulate microglial activity. Studies by Min et al. [58] have clearly documented the modulatory role of astrocytes to rescue reactive microglia from oxidative stress by inducing expression of heme oxygenase-1, thereby limiting microglial reactive oxygen species levels. It is also important to note that both PGE_2_ and conditioned medium from astrocytes have been shown to attenuate IL-12 cytokine release by activated microglia and thus inhibit Th1 immune responses in CNS autoimmune diseases [59]. In conclusion, nicotine treatment can not only control astrogliosis but may also have potential indirect effects on other activated brain cells such as microglia and thus provide dampening of excessive inflammation and damage.

## Conclusions

Reactive astrogliosis has been implicated in the disease progression of several neurodegenerative diseases such as Alzheimer’s disease, amyotrophic lateral sclerosis, Parkinson’s disease, and Huntington’s disease. Though both microglia and astrocytes contribute to disease pathology, their abundance and long-lasting role during the late stages of neuroinflammation make astrogliosis the obvious therapeutic target [60]. Cholinergic agonists could thus be clinically important in controlling neuroinflammation by reducing reactive astrogliosis and thereby delay neurodegeneration. Moreover, as we and others have shown the importance of prostaglandins in central cholinergic mechanisms, these data give implications that in addition to its well-documented peripheral effects on pain mediators, NSAIDs may also exert central nervous effects, also on central pain regulation, which demands further investigations in this context.

In summary, we have shown that activation of primary human fetal astrocytes with IL-1β results in reactive astrogliosis accompanied by the up-regulation of several proinflammatory cytokines. Treatment with nicotine inhibited not only cytokines such as IL-6, TNF-α, and IL-1β but also downregulated pivotal inflammatory mediators such as IL-8 and BuChE. Interestingly, the neuroprotective effects of nicotine were mediated by α7nAChR activation resulting in subsequent COX-2-dependent PGE_2_ production. These results confirm the potential for cholinergic neuroprotection and implicate an important role for the prostaglandin system in cholinergic regulatory effects, possibly important for the development of future therapeutic strategies of neuroinflammation.

## Abbreviations

ACh: Acetylcholine
cAMP: Cyclic adenosine monophosphate
CAP: Cholinergic anti-inflammatory pathway
CNS: Central nervous system
COX-2: Cyclooxygenase 2
cPGES: Cytosolic prostaglandin E_2_ synthase
DMSO: Dimethylsulfoxide
EAE: Experimental autoimmune encephalomyelitis
GFAP: Glial fibrillary acidic protein
IL-1β: Interleukin 1β
IL-6: Interleukin 6
LPS: Lipopolysaccharide
mPGES: Microsomal prostaglandin E synthases
nAChRs: Nicotinic acetylcholine receptors
NSAIDs: Non-steroidal anti-inflammatory drugs
PGE_2_: Prostaglandin E_2_
TGFβ: Transforming growth factor β
α7nAChR: Alpha7 nicotinic acetylcholine receptor
